# The SNP at −592 of human IL-10 gene is associated with serum IL-10 levels and increased risk for human papillomavirus cervical lesion development

**DOI:** 10.1186/1750-9378-7-32

**Published:** 2012-11-14

**Authors:** Kirvis Torres-Poveda, Ana I Burguete-García, Miguel Cruz, Gabriela A Martínez-Nava, Margarita Bahena-Román, Esmeralda Ortíz-Flores, Abrahan Ramírez-González, Guillermina López-Estrada, Karina Delgado-Romero, Vicente Madrid-Marina

**Affiliations:** 1 Dirección de Infecciones Crónicas y Cáncer. Centro de Investigación sobre Enfermedades Infecciosas (CISEI), Instituto Nacional de Salud Pública, Av. Universidad 655, Santa María Ahuacatitlán, Cuernavaca, C.P.62100, Cuernavaca, México; 2 Unidad de Investigación Médica en Bioquímica, Hospital de Especialidades, Centro Médico Siglo XXI, IMSS, Mexico, DF, Mexico; 3 Private Health Center for Gynecology, Cuernavaca, Morelos, Mexico; 4 Centro de Atención para la Salud de la Mujer (CAPASAM). (Center for Women’s Health), Health Services of the State of Morelos, Cuernavaca, Mexico

**Keywords:** IL-10 promoter polymorphisms, Squamous intraepithelial cervical lesions, IL-10 expression, Risk factors

## Abstract

**Background:**

Women with Human Papilloma Virus (HPV) persistence are characterized by high levels of IL-10 at cervix. We have determined whether polymorphisms of IL-10 gene promoter might be associated with increased risk of squamous intraepithelial cervical lesions (SICL) and whether exist significative differences of IL-10 mRNA expression at cervix and systemic and serum IL-10 protein between SICL cases and non-Cervical Lesions (NCL).

**Methods:**

Peripheral blood samples from SICL (n = 204) and NCL (n = 166) were used to detect IL-10 promoter polymorphisms at loci -592A/C (rs1800872), -819C/T (rs1800871), -1082A/G (rs1800896), -1352A/G (rs1800893), by allelic discrimination and to evaluate serum IL-10 protein. Cervical epithelial scrapings from NCL and biopsies from SICLs were used for HPV-typing and to evaluate IL-10 mRNA expression level. The systemic and local IL-10 mRNA expression levels were measured by real time-PCR. Genotypic and allelic frequencies of the selected polymorphisms were analyzed by logistic regression, adjusting by age and HPV-genotype, to determine the association with SICL.

**Results:**

No significant differences were found between genotype frequencies at loci −819, -1082, and −1352. Individuals carrying at least one copy of risk allele A of polymorphism −592 had a two-fold increased risk of developing SICL [adjusted odds ratio (OR), 2.02 (95% CI, 1.26-3.25), p = 0.003], compared to NCL. The IL-10 mRNA expression and serum IL-10 protein, were significantly higher in SICL cases (p < 0.01), being higher in patients carrying the risk allele A.

**Conclusions:**

The −592 polymorphism is associated with increased risk of SICL and can serve as a marker of genetic susceptibility to SICL among Mexican women. According to IL-10 levels found in SICL, IL-10 can be relevant factor for viral persistence and progression disease.

## Background

The majority of women clear HPV infection spontaneously by the antiviral immune response, but it has been suggested that persistence of HPV infection can be associated with the development of squamous intraepithelial cervical lesion (SICL) [1]. The fact that only a small proportion of HPV-infected individuals will eventually develop cancer of the cervix and the long latency period between primary infections and cancer emergence suggest that additional factors are involved in the progression. Other factors, such as genetic susceptibility or alteration of the immune response, increase the incidence of HPV-associated lesions [1].

A predominance of the Th2 cytokine profile, in association with a diminished Th1 profile, has been demonstrated in patients with cervical cancer (CC) [2-4]. This shift from Th1 to Th2 might be responsible for facilitating tumor progression by subverting various cellular immune surveillance mechanisms. An impaired cellular immune response induced by immune suppressor cytokines, such as Interleukin (IL)-10 and Transforming Growth Factor Beta (TGF-β1) has been involved in High Risk HPV (HR-HPV) persistence and CC development [2,3,5-7].

IL-10 is a Th2 anti-inflammatory cytokine that participates in the regulation of the immune response at several levels [8]. The primary T cell source of IL-10 is the T regulatory cell and Th1 and Th2 lymphocytes [9]. IL-10 is produced by activated CD4+ and CD8+ T cells, Epstein–Barr virus-transformed lymphoblastoid cell lines, fibroblasts and monocytes. It has potent inhibitory effects on T cell proliferation, inflammation and production of Th1 cytokines. Other sources of IL-10 include B lymphocytes [10], myeloid dendritic cells [11], and mast cells [12]. IL-10, prevents tumor antigen presentation to CD8+ T cells by suppressing the expression of Major Histocompatibility Complex (MHC) class I and II antigens [13], inhibits costimulatory activity of antigen-presenting cells by inhibiting CD3-Z chain expression [7], and down-regulates MHC class II expression on monocytes [14]. These properties support the role of IL-10 as a strong immunosuppressive cytokine. On the other hand, IL-10 overexpression impairs Th1 cytokine production with an absence of specific T cell activation in the tumor microenvironment, thus contributing to cancer development [4]. IL-10 mRNA has been found in a variety of freshly excised human tumors [15-21], including CC [4,6].

The biological significance of IL-10 production by tumor cells remains unexplored. Several studies have shown that the capacity for IL-10 production in individuals appears to be influenced by genetic makeup [22], and several groups have proposed IL-10 as a candidate gene for CC susceptibility [23-25].

Although several studies have reported that IL-10 expression might be associated with IL-10 gene polymorphisms, few studies have examined the association between the genotype frequency of IL-10 and the risk for SICL. In this paper, we investigated whether there is an association between IL-10 promoter polymorphisms and the risk for SICL in Mexican women, and furthermore, whether there is any variation in systemic IL-10 mRNA expression level and at cervix level, and the protein level in serum, in the SICL cases with respect NCL.

## Materials and methods

### Study design

A cross-sectional study was carried out including 204 newly diagnosed SICL cases (aged 18–70 years) recruited from Centro de Atención para la Salud de la Mujer del Estado de Morelos in Mexico, between June 2008 and November 2010. The SICL cases classified as low- and high-squamous intra-epithelial lesions (L/HSIL) were evaluated by two pathologists and only the confirmed ones were included. The whole population was Mexican Mestizo with a history of three previous generations being born in Mexico and with a residency period of > 1 year in the study area (inclusion criteria). Women without intraepithelial cervical dysplasia or cancer (n = 166), were selected as controls (aged 19–72 years) from Centro de Atención para la Salud de la Mujer del Estado de Morelos in Mexico between January 2008 and November 2010. They had class I or II Papanicolaou study negative to high-risk HPV infection and had a normal colposcopy. Also, to eliminate any selection bias, SICL cases and controls were screened to ensure that they do not have active infection Sexually Transmitted Disease (STD) and chronic inflammatory diseases during sampling, and used as an exclusion criterion.

The selected controls were matched to the cases by age (±5 years). The present study was approved by the Bioethics and Research Committee, all study subjects signed an informed consent letter and all investigation was carried out according to the Declaration of Helsinki. Each subject was interviewed for lifestyle, socio-demographic and hormonal factors known to be associated with increased risk of SICL.

### Specimens collecting and samples processing

Peripheral blood from all the subjects was collected by venipuncture, before any medical treatment, in vacutainer tubes (7 ml) containing EDTA (Becton Dickinson, BD, Franklin Lakes, NJ, USA) and in serum-separator vacutainer tubes (4 ml). Cervical epithelial scrapings cells and fresh cells biopsies obtained from women diagnosed with NCL and SICL, respectively. Peripheral blood mononuclear cells (PBMC) were obtained by Ficoll-hypaque density gradients (Hystopaque, Sigma Chemical Co). Genomic DNA was extracted from PBMC using Genomic DNA Purification Kit (Fermentas Life Sciences, Vilnius, Lithuania) and from cervical epithelial scrapings and biopsies previously digested with proteinase K. All possible measures were taken to avoid cross-contamination of samples during DNA extraction. DNA concentration and purity was evaluated by Thermo Scientific NanoDropTM 1000 Spectrophotometer (260/280) and the integrity of the DNA was determined by electrophoresis in agarose gels at 0.8%.

Total RNA was isolated from PBMC, cervical epithelial scrapings and biopsies, using the trizol reagent from Invitrogen. First-strand complementary (cDNA) synthesis was carried out according to protocol available at the following electronic page: http://es-mx.invitrogen.com/site/mx/es/home/References/protocols/nucleic-acid-amplification-and-expression-profiling/cdna-protocol/first-strand-cdna-synthesis-from-total-rna-or-poly-a-rna.html ]. Primers for the human housekeeping glyceraldehyde-3-phosphate dehydrogenase- GAPDH (250 pb) were used to verify cDNA integrity. For IL-10 gene expression analysis, real-time PCR amplification was performed by adding 2 μl of each cDNA sample to a final reaction mixture of 10 μl containing 5 μl of TaqMan PCR Master Mix for expression, 0.4 μl probe and 2.6 μl Dnase-free, molecular grade water. Amplification cycles (performed on a 7900HT Fast Real-Time PCR System from Applied Biosystems, Foster City, CA, USA) were: 94°C for 10 minutes, then 40 cycles at 94°C for one minute, 54°C for one minute, 72°C for one minute and 30 seconds; followed by 72°C for 15 minutes. GAPDH and HPRT1 (hypoxanthine phospho-rribosyl transferase) were used to normalize the amount of IL-10 mRNA present in each sample [26]. For the construction of the dynamic range curve for the analysis of IL-10 expression at the systemic level, PBMC was used, stimulated with phytohemaglutinin for 72 hours, as well as the SiHa cell line (which constitutively expresses IL-10) for the analysis of IL-10 expression at the cervix level.

Cervical epithelial scrapings and biopsy specimens were tested for HPV. Briefly, viral DNA fragments from the samples were amplified using consensus primers MY09/MY11 [27]; LIC1/LIC2 [28]; and GP5/GP6 [29]; that flanking the L1 region of HPV capsid (Table 1). PCR amplification of GAPDH (250 bp) was used as an internal control for DNA quality. Cell lines expressing HPV 16 (SiHa) and HPV-18 (HeLa) were used as positive controls, and deionized H_2_O as a negative control. All products were analyzed by electrophoresis in 2% agarose gels. The purified DNA band obtained was sequenced by the Sanger method. The HPV sequences were analyzed by BLAST. HPV types were categorized according to phylogenetic patterns into low and high risk, HPV16 and HPV18.

**Table 1:** Consensus primers used for testing HPV

**Gene**	**Primer sequence**	**Annealing temperature**	**Fragment size**	**Reference**
**MYO9/MY11**	5′-CGT CCM ARR GCA WAC TGA TC-3′ and 5′-GCM CAG GGW CAT AAY AAT GG-3′	57°C	450 bp	[27]
**L1C1/L1C2**	5′-CGT AAA CGT TTT CCC TAT TTT TTT-3′ and 5′-TAC CCT AAA TAC TAC TCT GTA TTG-3′	50°C	250 bp	[28]
**GP5/GP6**	5′-TTT GTT ACT GTG GTA GAT ACT AC-3′ and 5′-GAA AAA TAA ACT GTA AAT CAT ATT C-3′	40°C	150 bp	[29]

The collected samples of blood in serum-separator vacutainer tube were centrifuged to obtain serum and the IL-10 protein levels were determined using the human high sensitivity IL-10 ELISA kit (Abcam, Cambridge UK). All samples were measured in duplicate and the final IL-10 concentration was calculated as the average of duplicate readings. The results were expressed as picograms per milliliter (pg/ml).

### Genotyping

The Single Nucleotide Polymorphisms (SNPs) were selected with the following criteria: 1) Validated SNPs for frequency or for utilization in the HAPmap Project. 2) SNPs in the promoter region for which scientific evidence has been published on their potential role in transcriptional regulation of IL-10. For this selection, the Ensemble program was used, available at the following electronic page: http://www.ensembl.org/index.html. 3) SNPs in promoter IL-10, located in binding sites of transcription factors that potentially influence IL-10 transcriptional activity, reported in the following database: SNPper. URL: http//snpper.chip.org.

Six SNPs -2025A/G (rs1800892), -1352A/G (rs1800893), -1082A/G (rs1800896), -819C/T (rs1800871), -657A/G (rs1800895) and -592A/C (rs1800872) in the proximal promoter region of IL-10, were typed by allelic discrimination based on the assay of 5'-nuclease by the technology of the polymerase chain reaction, in combination with TaqMan fluorogenic probes in the Applied Biosystems 7900HT Fast Real-Time PCR System with a 384-Well Block Module. Reactions were performed in 5 μl volumes and contained 25 ng DNA, 1X TaqMan PCR Master Mix for genotyping (Applied Biosystems, Foster City, CA, USA) and 900 nM of each probe. Cycling conditions on the ABIprism 7900 HT (Applied Biosystems, Foster City, CA, USA) were 2 min, 50°C; 10 min, 95°C followed by 40 cycles of 15 sec, 92°C; and 1 min, 60°C. End-point fluorescence was measured immediately after cycling. All tests were done in duplicate and the alleles were assigned using SDS 2.1 software (Applied Biosystems). Quality control for the genotyping results was achieved with no sample controls, common homozygous and rare homozygous controls, in addition to retesting of samples with indeterminate results. The concordance between duplicate genotypes was greater than 90%; for quality control of the genotyping, we used a call rate of 0.99 for SICL and NCL (number of individuals assigned a genotype). When the call rate was less than 0.99, the DNA was reextracted; and new genotyping was done (only 6 samples required this procedure).

### Statistical analysis

Relevant variables between SICL and NCL, using *χ*^2^ or Kruskall Wallis test for categorical and continuous variables were compared, respectively. Hardy-Weinberg equilibrium was assessed for each SNP using the allelic frequencies of the control group. A logistic regression analysis was used to evaluate the association between SICL and the genotypes in codominant, dominant and recessive inheritance models, adjusting by potential confounders (age and HPV genotype). ORs were obtained with their respective 95% CI for each one of the genotypic variants. The reference variant was the homozygous of the most common variant. We used the Bonferroni method for correction of multiple comparisons. We accepted a probability of *α* = 0.05 for making at least one error and compared each SNP by three models of inheritance; we then divided our probability error three ways and used the cutoff: *α* = 0.05/4 = 0.012 = 0.01. The effect of having one or more risk-associated alleles with SICL was evaluated by multiple logistic regressions. All possible 2-way interactions between SNPs and between SNPs and IL-10 mRNA expression at the systemic level were tested by adding multiplicative terms in the multivariate logistic models.

IL-10 mRNA expression at the systemic level and in the cervix, as well as the analysis of IL-10 protein concentration at the serum level, across the groups, were analyzed using the Wilcoxon Mann–Whitney test. The mean estimated difference of Expression Relatives Units between SICL and NCL at the systemic level and in cervix was evaluated by lineal regression analysis adjusting by age and HPV genotype. To avoid stratification by ethnicity that could potentially confound genetic analyses, we confined our analysis to Mexican Mestizo population with two previous generations born in Mexico.

The power calculation was evaluated for each of the analyzed SNPs, taking into account the value of n fixed for the study and the minor allele frequency for each SNP in the NCL (P_1_), with an expected OR of 1.2, 1.5, 2, 2.5 and 3. The established alpha value was 0.05. The P2 calculation was performed using the following formula: P_1_*OR/(1-P_1_) + (P_1_*OR). The statistic power obtained for the SNP −592 C/A for OR 1.2, 1.5, 2, 2.5 and 3 was 0.34, 0.75, 0.99, 1, 1; respectively. All analyses were performed using STATA version 9.2 (StataCorp, Collage Station, TX, EUA) software program for Windows.

## Results

Socio-demographic and reproductive sexual characteristics of the studied population are presented in Table 2. SICL cases were, on average, older than NCL, they had the first sexual intercourse at a younger age, and also, a higher number of pregnancies (more than 3 pregnancies). There was a significant difference between the groups regarding the variables: age at first intercourse, socioeconomic level, number of pregnancies and history of STD (p = 0.0001). Models by logistic regression, adjusted for age and HPV genotype, confirmed the association between known reproductive and socio-demographic factors (first sexual intercourse, multiparity, socioeconomic level and history of STD) with SICL. The HPV prevalence in the studied population was 61%. The most prevalent genotype was HPV-16, followed by genotypes 18 and 59.

**Table 2:** General characteristics of the study population

**Characteristics**	**SICL cases (204)**	**NCL (166)**	**P value&**
Age (y) *			
Mean (SD)	36.3 (10.37)	35.8 (11.40)	0.42
Age at first sexual intercourse (y)*			
Mean (SD)	18 (3.07)	19.83(3.98)	**0.001**
Age at menarche (y)*			
Mean (SD)	12.79 (1.55)	12.88 (1.42)	0.57
**BMI (Kg/m**^**2**^)*			
Mean (SD)	26.02 (4.21)	25.14 (4.37)	0.06∞
**Socioeconomic Level (%)**+			
Low	**81.86**	21.08	**0.001**
Medium	18.14	78.92	
**Parity** +			
< 3	31	87.3	**0.001**
>3	**69**	12.65	
**Number of lifetime sexual partners** +			
<3	88.24	81.93	0.12
4 a 9	9.31	16.27	
>10	2.45	1.81	
**History of STD** +			
No	28.43	52.4	**0.001**
Other STDs	**55.39**	36.14	
HPV	16.18	11.45	
**Family planning method** +			
None	12.75	21.69	**0.001**
Hormonal Methods (6 months-5 years)	41.18	43.37	
Other methods	**46.08**	34.94	
**HPV genotype**+			**0.001**
Negative	29.9	38.55	
Other genotypes high risk	16.67	22.29	
HPV 18	7.84	21.69	
HPV 16	**41.67**	14.46	
HPV 16 and 18	3.92	3.01	

*SICL*: Squamous Intraepithelial Cervical Lesion.

*NCL*: Non-Cervical Lesion.

The results are expressed as means and SD for continuous variables.

Categorical variables are expressed in percentages.

& P value, Kruskall Wallis continuous variables* and *Χ*^2^ categorical variables+.

*P <* 0.05 NCL versus SICL.

∞*T* test for variables with normal distribution.

A descriptive analysis of selected polymorphisms was carried out (Table 3), establishing genotypic and allelic frequencies for the -592A/C, -819C/T, -1082A/G and -1352A/G in the SICL and NCL samples. A greater frequency was observed for the A/C genotype of the −592 polymorphism among SICL (50.3%) than among NCL (42%). No significant deviations were observed from HWE among controls in any of the genotyped SNPs. Table 3 shows the association analysis of the SNPs selected of IL-10 promoter region and the risk of SICL with ORs estimated through conditional logistic regression and adjusted by age and HPV genotype. Only C/A genotype of the −592 polymorphism had a significant association with an OR of 2.0 (95% CI, 1.21-3.32), p = 0.007 for the codominant model, confirmed in the dominant model A/C + A/A with an OR of 2.0 (95% CI, 1.26-3.25), p = 0.003. In assessing the association for alleles, a marginal association was found with an OR of 1.32 (95% CI, 0.97-1.81), p = 0.07. The other three SNPs showed no significant differences in any of the tested genetic models. The multiple models adjusted for age and HPV genotype did not lead to considerable changes in any of the results. When we performed the association analysis of SNP −592 C/A in the promoter region of the IL-10 gene with HPV infection, no statistically significant association was found for any of the models analyzed (data no show).

**Table 3:** Association analysis of SNPs (−592, -819, -1082, -1352) in the promoter region of the IL-10 gene, in Squamous Intraepithelial Cervical Lesions (SICL)

**(−592 C/A) (rs1800872)**				**(−819 C/T) (rs1800871)**			
**Genotype**	**n(%)SICL/(%)NCL (n = 204/166)**	**OR**a **(IC95%)**	p Value*	**Genotype**	**n(%)SICL/(%)NCL (n = 204/166)**	**OR**a **(IC95%)**	p Value*
**Codominant Model**				**Codominant Model**			
C/C	50(24.5)/66(40)	1		C/C	64(31.4)/57(34.3)	1	
C/A	105(52)/70(42)	**2.0(1.21-3.32)**	**0.007**	C/T	94(46)/75(45.2)	1.16(0.71-1.92)	0.53
A/A	49(24)/30(18)	**2.07(1.10-3.89)**	**0.02**	T/T	46(22.6)/34(20.5)	1.15(0.62-2.12)	0.64
**Dominant Model**				**Dominant Model**			
C/C	50(24.5)/66(40)	**1**		C/C	64(31.4)/57(34.3)	1	
C/A + A/A	154(76)/100(60)	**2.02(1.26-3.25)**	**0.003**	C/T + T/T	140(68.5)/109(65.7)	1.16(0.73-1.84)	0.51
**Recessive Model**				**Recessive Model**			
C/C + C/A	155(76)/136(82)	1		C/C + C/T	158(77.4)/132(79.5)	1	
A/A	49(24)/30(18)	1.37(0.79-2.39)	0.25	T/T	46(22.6)/34(20.5)	1.05(0.61-1.81)	0.84
***Alleles***				***Alleles***			
C	205(51)/202(60)	1		C	222(54)/189(57)	1	
A	203(49)/130(40)	1.32 (0.97-1.81)	0.07	T	186(46)/143(43)	1.10(0.80-1.50)	0.53
p HWE**			0.13	p HWE**			0.31
**(−1082 A/G) (rs1800872)**				**(−1352 G/A) (rs1800893)**			
**Genotype**	**n(%)SICL/(%)NCL (n = 204/166)**	**OR**a **(IC95%)**	p Value*	**Genotype**	**n(%)SICL/(%)NCL (n = 204/166)**	**OR**a **(IC95%)**	p Value*
**Codominant Model**				**Codominant Model**			
A/A	125(61.3)/92(55.4)	1		G/G	130(63.72)/102(61.44)	1	
A/G	66(32.3)/62(37.3)	0.95(0.59-1.52)	0.84	G/A	64(31.37)/53(31.94)	1.28(0.78-2.07)	0.31
G/G	13(6.4/12(7.3)	1.02(0.42-2.44)	0.96	A/A	10(4.9)/11(6.62)	0.85(0.33-2.22)	0.75
**Dominant Model**				**Dominant Model**			
A/A	125(61.3)/92(55.4)	1		G/G	130(63.72)/102(61.44)	1	
A/G + G/G	79(38.7)/74(44.6)	0.96(0.61-1.50)	0.87	G/A + A/A	74(36.27)/64(38.56)	1.20(0.76-1.90)	0.42
**Recessive Model**				**Recessive Model**			
A/A + A/G	191(93.6)/154(92.7)	1		G/G + G/A	194(95)/155(93.38)	1	
G/G	13(6.4/12(7.3)	1.04(0.44-2.44)	0.92	A/A	10(4.9)/11(6.62)	0.78(0.30-1.99)	0.6
***Alleles***				***Alleles***			
A	316(77)/246(74)	1		G	324(79)/257(77)	1	
G	92(23)/86(26)	1.01(0.71-1.45)	0.92	A	84(21)/75(23)	1.08(0.74-1.58)	0.65
p HWE**			0.72	p HWE**			0.26

a. Odds ratio adjusted by age and HPV genotype.

** Hardy-Weinberg Equilibrium in controls (NCL).

*p Value < = 0.01 adjusted by Bonferroni.

In order to explore whether the SNPs could act together to increase SICL risk, the allele load and tested the effect on risk for SICL were calculated (Table 4). We found that having one or more risk alleles had a statistically significant association with SICL, compared with having no risk alleles; p for trend = 0.014.

**Table 4:** Risk allele load and risk for Squamous Intraepithelial Cervical Lesions (SICL)

**Number of alleles associated with SICL**	**n**			**ORa.**	**CI 95%**	P Value*
	**Total**	**SICL**	**NCL**			
0	372	174	198	1	-	
1	280	127	153	1.07	0.78-1.47	0.66
2	38	17	21	1.17	0.58-2.33	0.65
≥3	50	14	36	**2.58**	1.33-5.00	**0.005**
**Trend p value**						**0.014**

n = Number of alleles.

a. OR adjusted by age and HPV genotype.

*p < 0.05.

**Figure 1:**
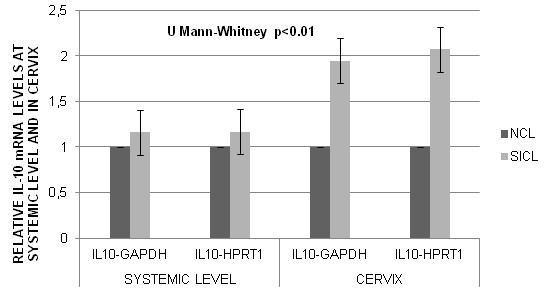
**IL-10 mRNA levels normalized to GAPDH and HPRT1 at the systemic level and in cervix, in NCL and SICL.** The levels were higher in SICL compared to NCL, at the systemic level and in cervix, p < 0.0001.

**Figure 2:**
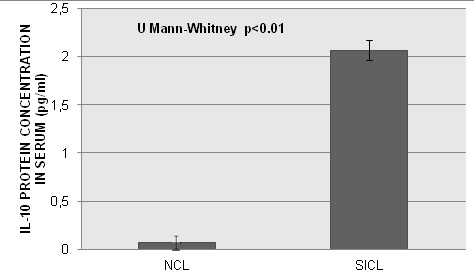
**IL-10 protein serum levels in women with SICL versus NCL.** The levels were higher in SICL compared to NCL, p < 0.0001.

IL-10 mRNA expression levels normalized to HPRT1 mRNA and GAPDH mRNA at the systemic level and in cervix of SICL and NCL patients are shown in Figure 1. The median levels of IL-10 mRNA relative to GAPDH and HPRT1 at the systemic level, were higher in patients with SICL than NCL and the difference was statistically significant (p < 0.01). IL-10 mRNA expression levels, normalized to GAPDH and HPRT1 in cervix, were higher in SICL compared to NCL, at the systemic level, p < 0.01 (Figure 1). In assessing the association between IL-10 mRNA expression levels at the systemic level and in cervix with SICL, the mean estimated difference of Expression Relative Units between SICL and NCL was 0.13 (95% CI, 0.09-0.18), p = 0.001 at the systemic level and 0.52 Expression Relative Units (95% CI, 0.44-0.59), p = 0.001 at the cervix level. Average IL-10 protein levels in serum were higher in SICL (2.06 pg/ml) compared to NCL (Figure 2). The mean estimated difference of IL-10 protein in serum, between SICL and NCL, was 1.86 pg/ml. When we evaluated the interaction between each of the SNPs: -592, -819, -1082 and −1352, and IL-10 mRNA expression at the systemic level, as well as the SNP-SNP interaction, no statistically significant interaction was found (data not shown).

## Discussion

The most prevalent HPV genotype in the analyzed population was 16, similar to other studies previously reported in Mexican population [30,31]. Persistent HPV infection, along with environmental and genetic factors such as IL-10, predisposes individuals to SICL and subsequent progression to cancer [2-4,6,32].

Previously, Hobbs et al., described a C to A exchange in the IL-10 promoter located 592 bp upstream from the transcription start site, which is located between putative consensus binding sequences for Sp1 (GGGGCGG), and a sequence with similarity to that recognized by members of the ETS family proteins (AGGAA). This −592 polymorphism is present in the promoter in a region with negative enhancer activity and is associated with loss of this activity [33]. Steinke et al., were the first ones that demonstrated that the C to A nucleotide exchange results in increased IL-10 gene promoter activity, supporting its role as a repressor element of the gene C variant [34]. Previous work from our group discovered that HPV-16 E6 and E7 oncoproteins binds to Sp1 transcription factor in the TGFß1 promoter and up regulates gene expression, through a GGGGCGG consensus sequence located at −180 to −172 of the TGFß1 human gene. Taken all together with the results of this paper, support the hypothesis that HPV-16 E6 and E7 oncoproteins may up regulate IL-10 gene expression [35].

This SNP has been linked with increased severity of a number of autoimmune diseases [36-39], and is associated with changes in tumorigenesis and transplantation tolerance [40], and rapid progression to AIDS in individuals infected with HIV [41,42]. The current study has shown that individuals homozygous for the A-allele of the IL-10 -592 polymorphism are at two times greater odds of having SICL [OR 2.07, (95% CI, 1.10-3.89), p = 0.02], as compared to NCL. A previous study carried out in 311 patients with cervical intraepithelial neoplasia (CIN), 695 cervical cancer patients, 115 family-based patients and 586 unrelated controls, in Caucasian population, revealed the same association, an increased risk for CIN (II–III) (OR 1.44 [1.06–1.97]) and squamous cell carcinoma of the cervix (OR 1.35 [1.04–1.75]) for individuals heterozygous for the A-allele of the IL-10 -592 polymorphism [25].

With respect to IL-10 mRNA expression at the systemic and cervix levels in patients with SICL and NCL, we also found that the level of IL-10 mRNA relative to GAPDH and HPRT1 in SICL was significantly higher than in NCL, at the systemic level. It has been shown that PBMC from patients with both SICL and CC produce higher levels of IL-10 following mitogenic stimulation, compared with control groups [2,7]. In addition, it has been shown that tumors can induce IL-10 production by PBMC [18,43]. Women with HPV infection have been found to have higher percentages of IL-10 positive T cells than healthy women, at the systemic level [44].

We also found that the level of IL-10 mRNA relative to GAPDH and HPRT1 in SICL was significantly higher than in NCL, at the cervix level. However, here we showed that IL-10 mRNA was undetectable in some cases with cervix without HPV infection, suggesting that normal cervical epithelium does not produce IL-10, similar findings had been found previously [4,5]. Abnormal IL-10 production at the cervix level has already been reported in patients with SICL and CC [6,45]. In CC, we report a high correlation between IL-10 immunostaining and the level of IL-10 mRNA expression in cervical biopsies, where there is a Th2/Th3 cytokine pattern expression, suggesting that HPV infection induces the transcription of immunosuppressive cytokines, IL-10 and TGF-ß, as a means to evade the host immune system [4,6,35].

In women with SICL, significant differences of the levels of IL-10 protein in serum were found between groups, with virtually undetectable serum protein in NCL women. The SICL presence, as well as the progression to CC has been associated with increased serum levels of IL-10 [6,44]. The level of IL-10 mRNA expression reported here, at the systemic level and in cervix, and protein level in serum are higher in SICL cases; this confirms our previous finding [6].

## Conclusions

We showed that individuals who are carriers of heterozygous IL-10 -592 C/A polymorphism had a two-fold increased risk of developing SICL (OR of 2.0 (95% CI, 1.21-3.32), p = 0.007, when compared to NCL, and to carry two copies of allele A confers two-fold increased risk of developing SICL (OR of 2.0 (95% CI, 1.10-3.89), p = 0.02. The SNP −592 C/A of the IL-10 promoter could be a risk factor for the development of HPV lesion in Mexican women, potentially associated with the production of high levels of IL-10 at systemic and cervix levels, which favors viral persistence and SICL development. Our study has several strengths: first, the cases studied here were precancerous lesions, where it has been demonstrated that IL-10 is responsible for the profound immune response alteration in HPV SICL [2,6]. Additionally, we previously demonstrate that there is a reduction of T-cell CD3-Z in patients with SICL, associated with IL-10 expression [7]. Second, the evaluation of IL-10 concentration was carried out in the systemic circulation and at the cervix level. Here, we demonstrated that there is a significant difference in the IL-10 levels in both sites (systemic and cervix) in SICL cases to compared with NCL. Therefore, it seems to be that the presence of IL-10 is an important factor in progressively impaired immune responses against HPV, allowing viral persistence and SICL development. Third, the −592 polymorphism associated with increased risk of SICL, can serve as a marker of genetic susceptibility to SICL among Mexican women and be considered as a candidate gene for genetic susceptibility devices that permit to identify women with high predisposition for the development of premalignant lesions in cervix that may become cases of cervical cancer if not are detected early.

## Abbreviations

SNP: Single Nucleotide Polymorphism; HPV: Human Papilloma Virus; SICL: Squamous intraepithelial cervical lesions; IL-10: Interleukin 10; mRNA: RNA messenger; NCL: Non-Cervical Lesions; PCR: Polymerase Chain Reaction; OR: Odds ratio; CC: Cervical cancer; TGF-β1: Transforming Growth Factor Beta; Th: T helper cells; MHC: Major Histocompatibility Complex; PBMC: Peripheral blood mononuclear cells; cDNA: First-strand complementary; GAPDH: Glyceraldehyde-3-phosphate dehydrogenase; HPRT1: Hypoxanthine phospho-rribosyl transferase; STD: Sexually Transmitted Disease; CIN: Cervical intraepithelial neoplasia.

## Competing interests

The authors declare that having no competing interests.

## Authors’ contributions

TPK participated in the design of the study, the collection of clinical materials, carried out the genotyping and expression analysis, the statistical analysis and drafted the manuscript. BGAI participated in the design of the study and the statistical analysis. CM participated in the analysis of the results. MNG participated in the genotyping analysis. BRM participated in the expression analysis. OFE and RGA participated in the processing of samples. LEG and DRK participated in the selection of study population, gynecological sampler and patient care. MMV participated in the analysis of the results and drafted the manuscript. All authors read and approved the final manuscript.
